# Assessment of Microbial Community Dynamics in River Bank Filtrate Using High-Throughput Sequencing and Flow Cytometry

**DOI:** 10.3389/fmicb.2018.02887

**Published:** 2018-11-29

**Authors:** Christina J. Fiedler, Christoph Schönher, Philipp Proksch, David Johannes Kerschbaumer, Ernest Mayr, Marija Zunabovic-Pichler, Konrad J. Domig, Reinhard Perfler

**Affiliations:** ^1^Laboratory of Microbiology, Institute of Sanitary Engineering and Water Pollution Control (SIG), Department of Water, Atmosphere and Environment, University of Natural Resources and Life Sciences, Vienna, Austria; ^2^Laboratory of Food Microbiology and Hygiene, Institute of Food Science, Department of Food Science and Technology, University of Natural Resources and Life Sciences, Vienna, Austria

**Keywords:** high-throughput sequencing, next generation sequencing (NGS), microbial community composition, drinking water quality, bank filtration, groundwater, absolute abundance, flow cytometry

## Abstract

Surface-groundwater interactions play an important role in microbial community compositions of river bank filtrates. Surface water contaminations deriving from environmental influences are attenuated by biogeochemical processes in the hyporheic zone, which are essential for providing clean and high-quality drinking water in abstraction wells. Characterizing the flow regime of surface water into the groundwater body can provide substantial information on water quality, but complex hydraulic dynamics make predictions difficult. Thus, a bottom up approach using microbial community shifting patterns as an overall outcome of dynamic water characteristics could provide more detailed information on the influences that affect groundwater quality. The combination of high-throughput sequencing data together with flow cytometric measurements of total cell counts reveals absolute abundances among taxa, thus enhancing interpretation of bacterial dynamics. 16S rRNA high-throughput sequencing of 55 samples among six wells in a well field in Austria that is influenced by river bank filtrate within a time period of 3 months has revealed both, clear differences as well as strong similarity in microbiome compositions between wells and dates. A significant community shift from April to May occurred in four of six wells, suggesting that surface water flow regimes do affect these wells stronger than others. Triplicate sampling and subsequent sequencing of wells at different dates proved the method to be reproducible. Flow cytometric measurements of total cells indicate microbial shifts due to increased cell counts and emphasize the rise of allochthonous microorganisms. Typical freshwater bacterial lineages (Verrucomicrobia, Bacteroidetes, Actinobacteria, Cyanobacteria, Armatimonadetes) were identified as most increasing phyla during community shifts. The changes are most likely a result of increased water abstraction in the wells together with constant river water levels rather than rain events. The results provide important knowledge for future implementations of well utilization in dependency of the nearby Danube River water levels and can help drawing conclusions about the influence of surface water in the groundwater such that hygienically save and clean drinking water with a stable microbial community can be provided.

## Introduction

Groundwater provides a vast reservoir for drinking water and contributes to the drinking water supply of 1.5 – 3 billion people worldwide (Katsanou and Karapanagioti, 2017). Its functioning is dependent on a versatile ecosystem that maintains clean and hygienically safe water via self-purification processes, implemented by diverse autochthonous microbial communities (Danielopol et al., 2003; Griebler et al., 2014). In order to protect this irreplaceable ecosystem, national as well as international strategies are developed to prevent pristine aquifers from vast anthropogenic impacts, possibly leading to permanent microbial turnover and thus resulting in degradation of water quality (Getches, 1989; Bredenhann and Braune, 2000; European Commission, 2000). Nevertheless, the definition of good groundwater quality only depends on physico-chemical parameters, but excludes microbiological characteristics (European Commission, 2000).

Groundwater ecosystems are characterized by oligotrophic environments containing highly adapted microorganisms due to low nutrient availability and further abiotic factors (Karczewski et al., 2017). As a consequence, the total number of microorganisms in groundwater is 10 – 100 times lower as compared to surface water (Pedersen, 2000; Griebler and Lueders, 2009) with a large proportion belonging to the viable but non-culturable (VBNC) organisms (Roszak and Colwell, 1987). The vast majority of bacteria contributing to pristine water communities has not been cultured yet, thus bacterial physiology among them and metabolic community functioning remain widely unknown (Rappé and Giovannoni, 2003; Raes and Bork, 2008). Therefore, the investigation of changes among communities rather than the characterization of single samples can provide insights into adaptations of microbial communities to biotic as well as abiotic factors, such as temperature, turbidity, pH, dissolved oxygen, total and dissolved organic carbon and ammonia (Kaevska et al., 2016; Hou et al., 2018; Liao et al., 2018).

The influences that contaminations can have on microbial communities have been thoroughly studied (Li et al., 2017; Zhang Y. et al., 2017; Romera-Castillo et al., 2018; Wang et al., 2018), but knowledge on commonly occurring impacts investigated by microbial community analyses remain scarce (Pronk et al., 2009; Schwab et al., 2017).

Wells serving as drinking water resources in the vicinity of rivers are influenced by river bank filtration. Especially the hyporheic zone between surface water and groundwater provides living space for a variety of microorganisms and biological activity within this zone contributes to the natural attenuation of contaminants from surface water infiltrating into the aquifer, usually resulting in minor changes in the biogeochemical processes of the reservoirs (Boulton et al., 1998; Hiscock and Grischek, 2002). Although persistence of allochthonous microorganisms in the groundwater bodies is unlikely (Pronk et al., 2009), river bank filtration systems are considered to be vulnerable to extreme weather conditions such as floods and droughts (Sprenger et al., 2011; Besmer et al., 2017). Increased water level differences between river and wells due to season and rain events as well as increased pumping rates at well sites shorten interstitial residence time of water and reduce bank filtration processes, resulting in increased entry of allochthonous microorganisms in the groundwater body (Sprenger et al., 2011).

Investigating microbial differences between wells can indicate the degree of surface water influence among them (Lin et al., 2012). With such information at hand, conclusions can be drawn on distribution patterns of surface water within the well field as well as the period of the autochthonous microbial community to recover from allochthonous impacts. Furthermore, the detection of certain microbial groups can indicate both hygienic contamination as well as technical issues that may arise within the subsequent water treatment and distribution processes (Ascott et al., 2016).

So far, both, models and on-site investigations have been made to draw conclusions about impacts of surface water on the groundwater body and biological as well as physico-chemical parameters have been investigated within them (Ray et al., 2002; Wett et al., 2002; Ascott et al., 2016). Nevertheless, how entire microbial communities actually respond to events influencing bank filtration processes and how the distribution patterns of surface water in a well field can influence them, still needs to be investigated.

Within the last two decades, the development of high-throughput sequencing techniques has substantially increased our understanding of microbial population diversities in the environment and allows for the accurate identification of microbial taxa (van Dijk et al., 2014). However, the massively parallel sequencing techniques of hypervariable regions of 16S rRNA genes are associated with several biases that are partially overcome by optimized extraction methods, increased sequencing depth, improved bioinformatic tools, choice of primers as well as the use of replicates (Robasky et al., 2014; Hugerth and Andersson, 2017). So far, replicates have been used for DNA extraction and PCR amplification, respectively (Vivien et al., 2016; Ge and Yu, 2017), but samples were almost exclusively pooled prior to sequencing, thus making comprehensive evaluations of replicates impossible (Staley et al., 2015).

The application of flow cytometry for cell counting in water provides a useful tool to quantify entire bacterial communities (Van Nevel et al., 2017). Due to high sampling frequencies and rapid processing, short-term microbial dynamics, e.g., after precipitation events can be detected. The combined use of total cell counts together with sequencing data provides sample-specific estimated absolute taxon abundances (EAA). Due to the identification of real abundance changes, EAAs tend to be more informative regarding community population dynamics rather than relative abundances without absolute quantification (Zhang Z. et al., 2017). The goal of this study was to compare microbial communities within six wells that are characterized by river bank filtration. The investigation period included two seasons, winter and spring and covers water level differences between surface water and groundwater as well as a flood event. Using high-throughput 16S rRNA sequencing methods, microbial community compositions were investigated. Triplicate sampling was performed in order to assess reproducibility of the method. Spatial and temporal variation in microbial communities provide a basis for predicting both hygienically relevant as well as technical aspects of drinking water catchment.

## Materials and Methods

### Study Site and Sample Collection

**FIGURE 1:**
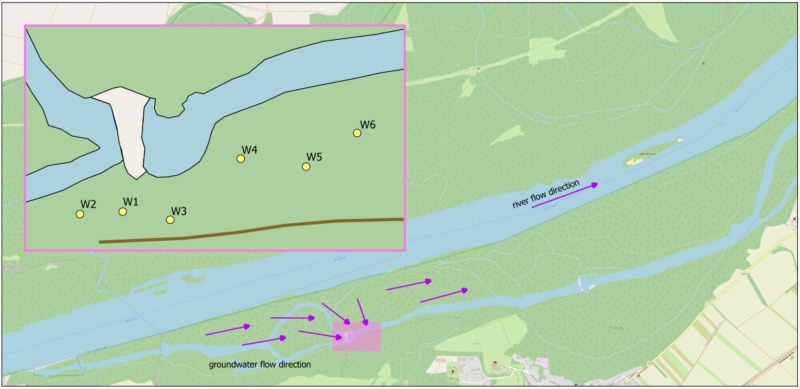
Outline map of the investigated well field. Arrows indicate the flow path. W1–W6, wells 1–6; MW, monitoring well of backwater.

The well field of sampling is located next to an intermittently donated backwater (alluvial part of the river with little current), at approximately 300 m distance from the Danube River, situated in the Donau-Auen National Park, Austria (Figure 1). Wells 1 – 4 and 6 are about equidistant from water (30–37 m), well 5 is 65 m away from water. A total of 55 samples from 6 different wells were taken. Samples were taken five times within 4 months, from February to May 2017. 15 samples at three different dates were taken as triplicates, as well as 10 single samples (depending on available resources). A sample volume of 5 L was taken in glass bottles covered with aluminum foil and muffled at 550°C for 5 h. For cell counting, 50 ml were additionally sampled in sterile centrifuge tubes (VWR, United States). Additional parameters were measured on-site (temperature, electrical conductivity, redox potential, water levels and abstraction rates in the wells). Sampling taps were thoroughly flamed and water was flushed for 10 min before sampling. Samples were transported and stored at 4°C and processed within 24 h.

### Filtration

Samples were filtered through sterile 0.2 μm PES membrane filters (47 mm Supor 200, Pall, MI, United States) using a water jet pump, rolled up using sterile forceps and transferred into collection tubes from the subsequently used extraction kit. Membrane filters were stored at -80°C until DNA extraction.

### Total Cell Count

The count of total cells was measured by fluorometric staining of nucleic acids and subsequent detection of cells with a flow cytometer based on the Austrian protocol (Zunabovic-Pichler et al., 2018). 297 μl of well mixed sample were stained with 3 μl SYBR^®^Green I (Life Technologies, Eugene, OR, United States), previously diluted 1:100 with DMSO (Fluka, Switzerland) and subsequently incubated in the dark for 13 min. Flow cytometric measurements were performed using a BD Accuri C6 flow cytometer (BD Life Sciences, CA, United States) containing a 50 mW laser emitting at a wavelength of 488 nm. The contained volumetric counting hardware was set to measure the number of particles in 50 μl, which is subsequently calculated to a volume of 1 ml. Measurements were implemented three times at a flow rate of 35 μl/min. Obtained data were processed using the R package flowCore. The bacterial signal was differentiated from background noise by electronic gating (Supplementary Figure 1).

### DNA Extraction

DNA was extracted from the filters using the DNeasy PowerWater Kit (Qiagen, Germany) following the protocol, with some modifications: Bead beating was extended to 10 min, followed by 4 min centrifugation at 4,000 *g*. The incubation time at 4°C after addition of inhibitor removal solution (IRS) was extended to 10 min. DNA was eluted in 50 μl elution buffer. Subsequently, DNA concentration was measured on a Qubit 2.0 fluorometer using a Qubit^®^dsDNA HS Assay Kit (Invitrogen, OR, United States) following manufacturer instructions. 1 μl DNA was added to 199 μl working solution for measurement. Genomic DNA as well as all further DNA products were stored at -20°C until processing.

### PCR Amplification and Sequencing

A two-step PCR barcoding approach was used as suggested by Herbold et al. (2015). For the first PCR reaction, generating the 16S amplicon products, the universal primers S-D-Bact-0341-b-S-17 (5′-CCT ACG GGN GGC WGC AG-3′) and S-D-Bact-0785-a-A-21 (5′-GAC TAC HVG GGT ATC TAA TCC-3′), targeting the hypervariable V3/V4 region of the 16S rRNA gene were used as described by Klindworth et al. (2013). PCR reactions were implemented using a reaction volume of 26 μl containing 12.5 μl Mastermix (KAPA HiFi Hotstart ready mix, peqlab, Germany), 5 μl forward and reverse primer (2 μM) each and 3.5 μl template DNA. The following PCR conditions were used: initial denaturation at 95°C for 5 min, followed by 32 – 37 cycles of denaturation at 95°C for 30 s, annealing at 66°C for 30 s and extension at 72°C for 30 s and a final extension step at 72°C for 5 min. The amount of cycles was adjusted to the initial DNA concentrations, ranging from 3.4 to 66.8 ng/μl. With each PCR reaction a negative control containing pure water (Sigma-Aldrich, Switzerland) as template was included to check for contaminations. PCR products were purified using the peqGOLD cycle-pure kit (Safety line, peqlab, Germany) according to manufacturer instructions with the following modifications: The drying step of the column after washing was extended to 5 min, 40 μl elution buffer were incubated for 5 min, followed by a 2 min centrifugation step. After amplification, a gel electrophoresis with a 100 bp ladder was performed on a 2% agarose gel to verify the size quality of purified PCR products. DNA was quantified using Qubit^®^. Concentrations ranged from 0.71 to 11.0 ng/μl.

The second PCR (Index PCR) converts amplicon products into library products for sequencing. It was implemented using Illumina primers from Nextera^®^XT index kit v2 (Illumina, CA, United States) and KAPA HiFi Hotstart ready mix. 5 μl of one N7xx and one S5xx primer (unique combination for each sample), 25 μl mastermix and 15 μl amplicon PCR product (normalized to 20 ng using purified water) were processed using the same PCR conditions as before, except of using only seven amplification cycles. Purification, gel electrophoresis and quantification of DNA was implemented as before. DNA concentrations ranged from 8.18 to 33.2 ng/μl. All samples were normalized to 12 ng/μl (for concentrations < 12 ng/μl undiluted PCR products were used) using 10 mM TRIS buffer (Sigma-Aldrich, Switzerland) and an aliquot of 5 μl was pipetted into the final pool. From this, a gel electrophoresis and a DNA quantification were implemented as a final quality control and the library was sequenced at Microsynth, Switzerland on an Illumina MiSeq platform, using the v3 reaction kit, 2 × 300 bp for paired-end sequencing. Demultiplexing and trimming of Illumina adaptor residuals, trimming of locus specific adaptors (cutadapt v 1.8.1) and merging of forward and reverse reads (Usearch v 8.1.1861) was carried out by Microsynth. Sequence data was received as.fastq files. The dataset can be found in the European Nucleotide Archive (ENA) PRJEB28172.

### Data Analysis

All data processing was performed using the Quantitative Insights into Microbial Ecology 2 (QIIME 2) 2018.4.0 microbiome analysis pipeline^1^ and R Statistical Software Version 3.44. The R package phyloseq was used to generate bar charts depicting relative and absolute abundance of taxa (McMurdie and Holmes, 2013). Using the DADA2 pipeline (Callahan et al., 2016), quality filtering, denoizing, merging and removal of chimeric sequences was applied on the data set. Taxonomies were assigned to the feature table using the naïve Bayes classifier implemented in QIIME 2 together with the SILVA 132 database (99% clustering, 7 levels, majority vote). Reads from triplicate samples were pooled for analyses of merged triplicates.

Generated total cell counts from flow cytometric data were multiplied by the relative abundances of taxa to reveal EAA among them as suggested by Props et al. (2017).

Alpha-diversity was investigated using Shannon-diversity index, Pielou’s evenness and observed richness parameters. Rarefaction was implemented for alpha-diversity measures. Principle Coordinate Analysis (PCoA) plots were generated from Bray-Curtis distances based on sequence variants to visualize beta diversity. Permutational Multivariate Analysis of Variance (PERMANOVA) based on Bray-Curtis dissimilarities (relative abundances) was performed to test for differences in beta-diversity among groups (Anderson, 2008). As implementation of PERMANOVA the function adonis of R-package vegan (Oksanen et al., 2018) was used, the number of permutations was set to 10,000.

Using the package DAtest (Russel et al., 2018) an optimal procedure for differential abundance testing was identified for both, relative abundances as well as EAAs. In short, DAtest finds well suited methods for the specific dataset at hand by repeated calculation of quality scores based on shuffling predictor variables, random spiking of features corresponding to the shuffled predictor variables and the application of a wide range of available testing methods aiming at identifying the spiked features. After application of DAtest the best scoring testing methods were used to find differentially abundant phyla.

## Results

### Bacterial Diversity

A total of 13,584,552 past filter reads (sum of forward and reverse reads) with a mean read length of 299 bp and a mean quality score of 31 were retrieved from 55 samples. After processing with QIIME2, library size ranged from 20,658 to 107,485 reads with a mean of 53,068 merged reads per sample (43% reads passing quality filtering). Since the number of reads per sample was considered sufficient for downstream analyses, no relaxation of quality filtering criteria was performed. 34,980 sequence variants were observed and used for downstream taxonomic analyses.

The Shannon alpha-diversity index ranged from 5.08 to 7.35. Observed richness and Pielou’s evenness ranged from 1344 to 4537 and 0.65 to 0.92, respectively. Well 4 represents the highest range between samples for all parameters (6.34 ± 0.77; 2,444 ± 728 and 0.82 ± 0.1, respectively) (Table 1).

**Table 1:** Alpha-diversity parameters and library size among wells.

	Shannon			Observed richness			Pielou’s evenness			Library size		
**Well**	**Mean ± *SD***	**Min**	**Max**	**Mean ± *SD***	**Min**	**Max**	**Mean ± *SD***	**Min**	**Max**	**Mean ± *SD***	**Min**	**Max**

**1**	6.70 ± 0.14	6.84	7.10	3,329 ± 1,017	2,277	4,306	0.87 ± 0.05	0.82	0.92	125,761 ± 71,506	53,917	196,925
**2**	6.99 ± 0.43	6.51	7.34	3,460 ± 938	2,405	4,198	0.86 ± 0.06	0.79	0.92	119,378 ± 55,911	54,848	153,358
**3**	6.85 ± 0.50	6.20	7.35	3,065 ± 1,255	1,649	4,537	0.86 ± 0.07	0.76	0.91	112,774 ± 77,875	37,341	198,58
**4**	6.34 ± 0.77	5.08	7.03	2,444 ± 728	1,344	3,34	0.82 ± 0.1	0.65	0.90	124,608 ± 80,534	34,21	241,095
**5**	7.19 ± 0.18	6.88	7.31	3,361 ± 1,017	1,895	4,239	0.89 ± 0.02	0.87	0.91	115,397 ± 50,687	46,724	163,021
**6**	6.76 ± 0.22	6.57	7.11	2,717 ± 801	1,874	3,672	0.86 ± 0.03	0.82	0.89	106,446 ± 38,955	60,551	142,871

*SD, standard deviation; Min, minimum; Max, maximum.*

### Triplicate Similarity

**FIGURE 2:**
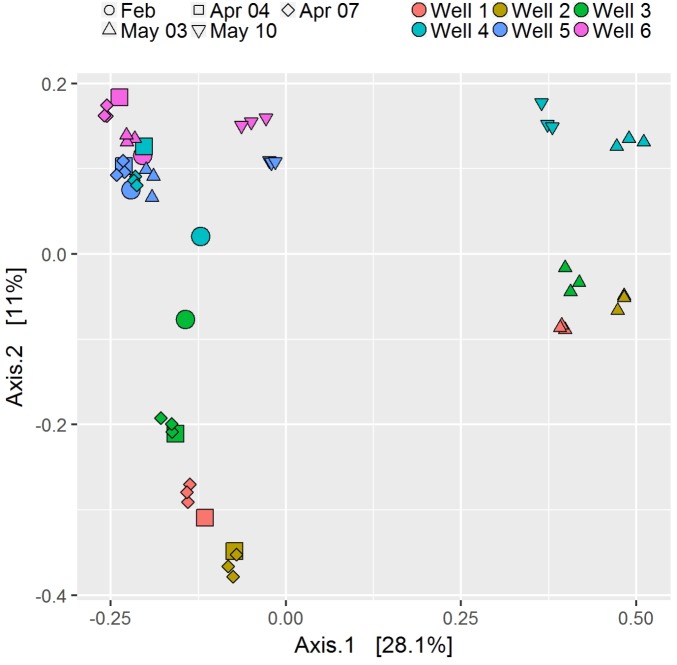
Principle coordinate analysis based on Bray–Curtis dissimilarity of all samples. Triplicates are shown as smaller symbols.

According to the suggestion of Staley et al. (2015), 15 of the investigated probes were sampled and processed in triplicates in order to compare diversity parameters. Principal coordinate analysis (PCoA) of triplicates showed similarity among triplicates compared to distinctiveness of samples (Figure 2). Shannon-diversity and Pielou’s evenness show a variation coefficient of 0.028 ± 0.021 and 0.010 ± 0.014, respectively within triplicates, whereas observed richness shows a higher variation of 0.171 ± 0.115 as well as variability in library size (variation coefficient 0.241 ± 0.163).

### Spatial and Temporal Variability

Well 5 shows to be most stable throughout the investigation period regarding both alpha-diversity (Shannon alpha-diversity index 6.88 – 7.31) as well as total cell counts (1.5 × 10^5^ – 1.8 × 10^5^ cells/ml), whereas well 4 is very variable in both parameters (5.08 – 7.03 and 1.3 × 10^5^ – 4.7 × 10^5^ cells/ml, respectively). Alpha-diversity among wells appears similar from February to April. Between April 4 and April 7, respectively and May 3 differences in diversity become more pronounced.

**FIGURE 3:**
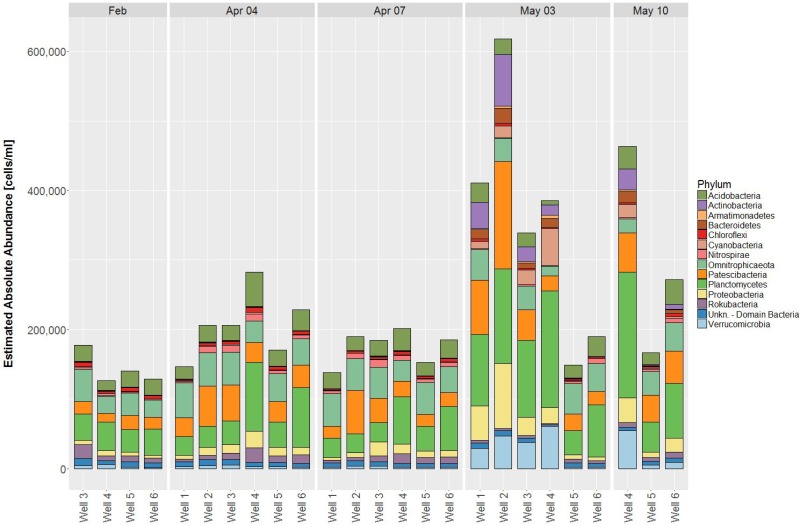
Estimated absolute abundances of phyla at the respective dates (cut-off value 1%). Reads from triplicate samples were pooled to one sample each.

Based on Bray-Curtis dissimilarity, the dynamics in community compositions of wells 1 – 3 behave in a similar way during the investigated period: A major shift occurs from April 7 to May 3, but wells shift in the same direction (Figure 2). Wells 4 – 6 also behave similar to each other with the exception that wells 5 and 6 do not show major shifts from April to May 3, but rather on May 10, although to a much lesser extent than well 4 (Figure 2). This is in general accordance with the geographical adjacency of wells 1 – 3 and 4 – 6, respectively (Figure 1), although spatial differences contribute less to the dissimilarity than temporal shift does [*y*-axis of PCoA accounts for only 11.0%, *x*-axis for 28.1% of dissimilarity (Figure 2)]. Microbial community changes correlate with the change in total cell counts: From May 3, cell counts increase in wells 1 – 4, well 6 shows that increase on May 10, but again to a lesser extent, well 5 remains similar (Figure 3). Considering that the most noticeable changes happen from April 7 to May 3 and that triplicate results from all wells exist among those dates, taxonomical identification will mainly focus on the dynamics between the two dates.

### Microbial Community Distribution

**FIGURE 4:**
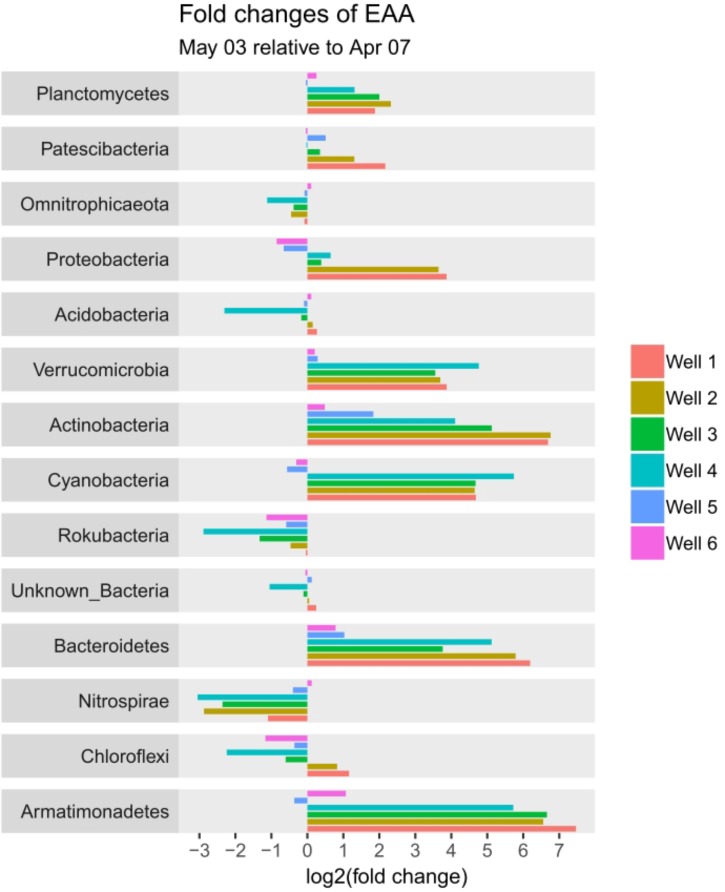
Fold-change plot of phyla from April 7 to May 3 based on estimated absolute abundances (EAA).

56 different phyla were identified, of which the most abundant phyla included Planctomycetes (12.2 – 48.2%), Omnitrophicaeota (2.6 – 34.1%), Patescibacteria (4.5 – 37.5%), Acidobacteria (1.3 – 17.9%) and Proteobacteria (2.2 – 16.2%) contributing to more than 75% relative abundance among all samples. Temporal dynamics in microbial communities contribute greatly to the variability of relative abundances: The most increasing phyla from April 7 to May 3 are Verrucomicrobia, Actinobacteria, Cyanobacteria, and Bacteroidetes, whereas Nitrospirae decrease simultaneously (Supplementary Figure 2). Considering total cell counts, microbial community distribution can be compared in EAA, whereby increasing phyla show a much larger impact due to increase in cell counts on May 3 and decrease of phyla diminishes, respectively (Figure 4).

Based on the outcomes of DAtest (Supplementary Figures 3, 4) a differential abundance testing of phyla between April 7 and May 3 in wells 1 – 3 (paired testing using wells) was conducted. The comparison of relative abundances based on LIMMA log (Ritchie et al., 2015) shows to be the most conservative with a total of 9 phyla being significantly different (*p* < 0.05). EdgeR quasi likelihood2 (Robinson et al., 2010) and LIMMA voom (Law et al., 2014; Ritchie et al., 2015), which score slightly better in the DAtest analysis, find more significant differences (12 and 11 phyla, respectively). Since the outcomes of DAtest show a considerable increase of false discovery rates (FDR) and false positive rates (FPR) for all the mentioned methods, especially in the case of a high number of spiked features (Supplementary Figure 3), the results must be considered cautiously.

The comparison of EAAs with the best scoring method LIMMA log shows significant differences (*p* < 0.05) for the phyla Actinobacteria, Armatimonadetes, Bacteroidetes, Cyanobacteria, Nitrospinae, Nitrospirae, Planctomycetes and Verrucomicrobia. In contrast to the testing of relative abundances, DAtest does not indicate an increase of FDR and FPR for all settings tested (Supplementary Figure 4).

The significant difference in Armatimonadetes is due to absence of the phylum in April, nevertheless abundances are low in May. The phyla Omnitrophicaeota and Acidobacteria (exception for well 4) seem to decrease when looking at relative abundances. In fact, they proofed to be stable, with little decrease or increase in EAA (Figure 4).

### Diversity Patterns Among Families

**FIGURE 5:**
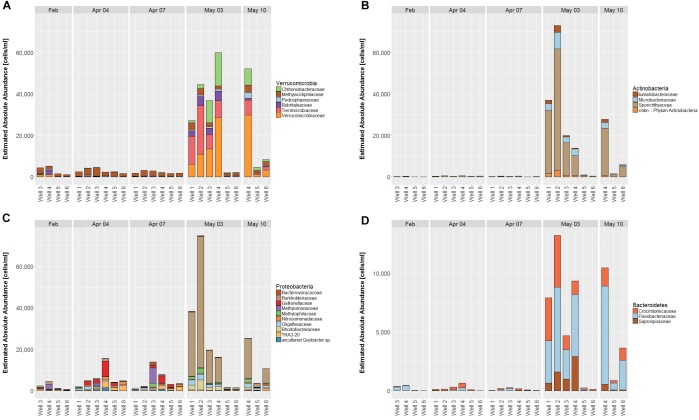
Estimated absolute abundance on family levels (cut-off value 0.5%) for the Phyla Verrucomicrobia **(A)**, Actinobacteria **(B)**, Proteobacteria **(C)**, and Bacteroidetes **(D)**. Due to lower abundance, *y*-axis scaling is smaller for **D** than for **A–C**.

The families Verrucomicrobiaceae and Terrimicrobiaceae contribute most to the increase in Verrucomicrobia from April to May, Methylacidiphilaceae is the most abundant family before May and counts remain similar throughout all dates (Figure 5A).

Among Actinobacteria, Sporichthyaceae is the most abundant family before and after the shift, however, overall counts are close to zero in April, suggesting that abundance of the entire phylum develops from surface water entry (Figure 5B).

Proteobacteria show diverse families as well as cell counts in April, which are predominantly replaced by Burkholderiaceae, accounting for most Proteobacteria in May (Figure 5C).

Among the Bacteroidetes, the families Crocinitomicaceae, Flavobacteriaceae and Saprospiraceae contribute exclusively to the phylum, of which Saprosphiraceae rises in May, the others are already abundant before, but to a lesser extent (Figure 5D).

### Phylum Based Sample Clustering

**FIGURE 6:**
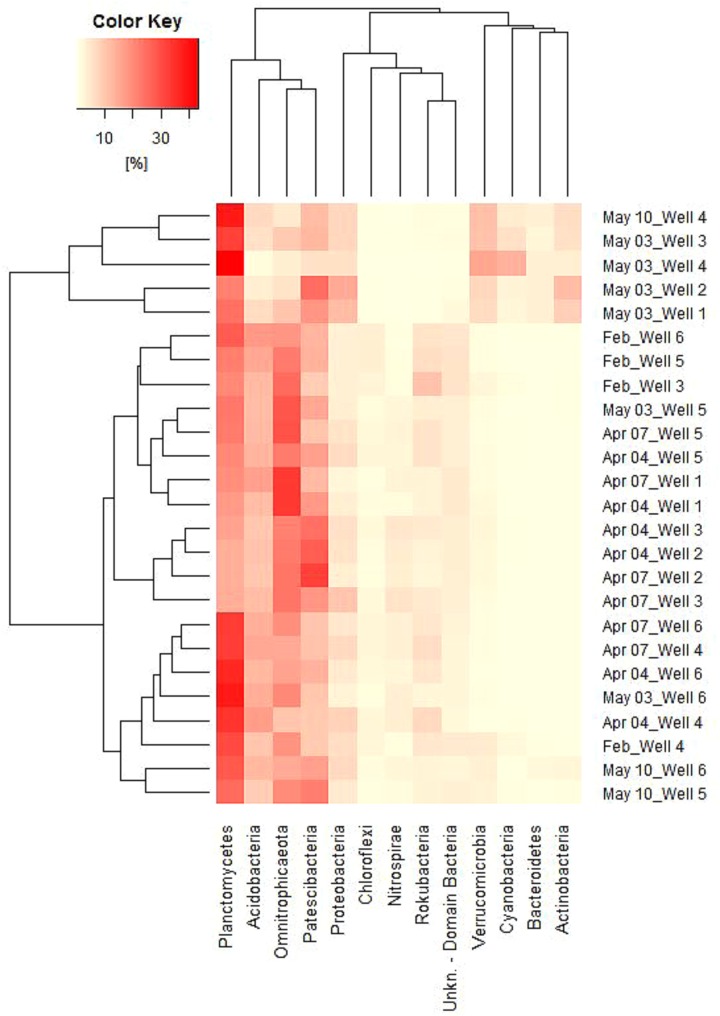
Heatmap of relative abundances of 13 phyla among all samples (cut-off value 2%). Vertical grouping based on sample similarity, horizontal grouping in dependency of Phylum abundance similarity.

As suggested before, the alignment of samples depending on both dates and wells based on EAA seems obvious (Figure 3). In dependency on relative abundances among all samples, certain groups can also be revealed, which are shown in Figure 6. Between samples, two groups are formed, of which one contains the samples from May 3, wells 1 – 4 as well as May 10, well 4. Regarding Phyla, three groups are formed, of which the first contains the four most abundant Phyla (Planctomycetes, Acidobacteria, Omnitrophica, and Patescibacteria), the second contains five Phyla (Proteobacteria, Chloroflexi, Nitrospirae, Rokubacteria, and an unknown bacterium phylum) and the third group contains the four most increasing phyla in May (Verrucomicrobia, Cyanobacteria, Bacteroidetes, and Actinobacteria). Within the second group, Proteobacteria represents the only phylum that remains rather stable between sample groups, whereas all other phyla strongly decrease in May as opposite to group 3, in which phyla increase (Figure 6).

### Water Level Differences

**FIGURE 7:**
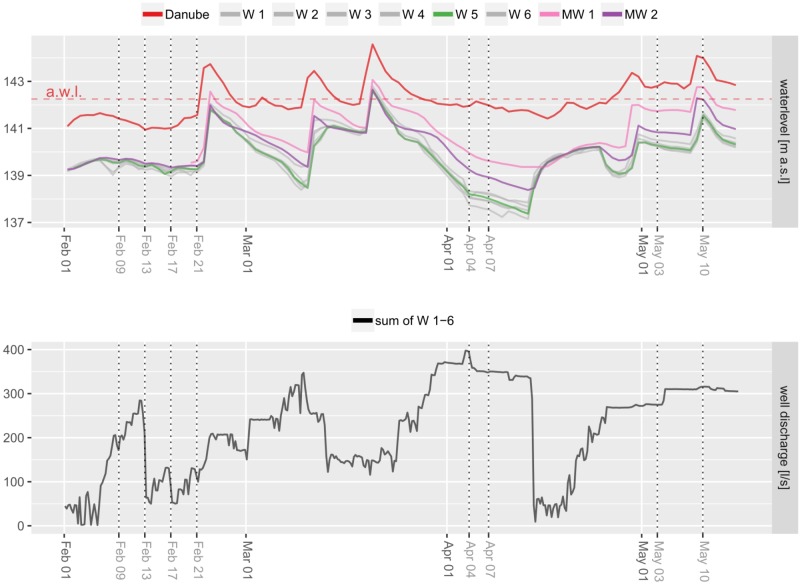
Water levels of wells and the Danube River. Differences in wells are due to pumping activities. Dotted vertical lines indicate sampling dates.

Danube River water levels (measured approx. 3 km upstream) range from 141 to 145 m above sealevel. Water levels among wells show high similarity regarding both, overall water levels as well as shifting patterns throughout the investigation period (Figure 7). Two monitoring wells in the backwater show water levels similar to the wells, but are characterized by slightly higher values during intensive abstraction in the wells, when well water levels decrease. In general, Danube, backwater and wells behave similar regarding water level shifts. A major difference between wells and Danube River develops during constant surface water levels over a stable period from end of March until end of April and simultaneous high water abstraction in the wells (Figure 7). Microbial community shifts indicate this period to be decisive for changing water compositions in wells 1 – 4. The investigated flood event on May 10 did not affect microbial communities immediately. Comparison of beta diversity between May 3 and May 10 in wells 4 – 6 was not significant (PERMANOVA *p* > 0.05).

### Abiotic Factors

It situ parameters have been measured during sampling (Supplementary Figures 5, 6). Noticeable is the electrical conductivity that is higher in well 5 as compared to other wells (846.8 ± 96.7 μS/cm in well 5 and 572.8 ± 57.3 μS/cm in all other wells, received from sampling dates April 7 and May 3). Conductivity in the Danube River and the backwater are lower with 445.5 ± 23.6 μS/cm and 430.8 ± 8.6 μS/cm, respectively.

## Discussion

The Donau-Auen National Park is characterized by small-scale natural heterogeneity due to former floods and rearrangements. Great differences in permeability, organic matter content and composition of the soil can influence subsequent groundwater characteristics. To investigate small-scale spatial as well as temporal changes, the microbial community patterns of 6 wells in a well field characterized by groundwater influenced by river bank filtration were investigated to reveal information about changes in microbial communities among wells. With such information at hand, conclusions can be drawn on the different impact of surface water on the well water and how this might contribute to optimized subsequent drinking water treatment.

### Triplicate Sampling

Cao et al. (2002) suggests the use of autosimilarity (average similarity among replicates) as a standardization method rather than sample size, as total taxon richness can vary across sampling sites. Therefore, standardized sample size is biased for compositional comparisons and ignores how complete communities are represented. The application of replicate samples instead can be used as a measure of sample community representativeness and provides an accurate predictor of richness. Furthermore, Wen et al. (2017) showed that community variation is larger for biological triplicates (i.e., triplicate sampling and subsequent separate preparation) than for technical triplicates (one sample, triplicate amplification). Both increase sampling effort and enhance reproducibility. Additionally, triplicate sampling provides a comparison of variety measurements of different, but still similar samples, thus allowing to discriminate between them.

The similarity within triplicates based on both, Bray-Curtis dissimilarity as well as low variation coefficients (alpha-diversity) shows that the sampling method reveals reliable results. Due to varying read counts among triplicates, which are shown in library size variations, observed richness parameters vary stronger than Shannon-diversity and Pielou’s evenness indices. Overall high read counts add confidence to reliable, high quality results, although high rejection rates of reads (57%) as a consequence of high stringency quality filtering decrease library size. Pooling of multiple libraries increases sequencing depth and consequently generates profound information on microbial communities (Zaheer et al., 2018), thus suggesting that triplicate sampling increases overall richness due to increase in library size and provides an important tool as proof of reproducibility. Furthermore, high sample volumes of 5 L account for less sequence variation (Staley et al., 2013). With decreasing costs in massive parallel sequencing technologies, we suggest replicate sampling (duplicates or triplicates) to become a basic requirement in future investigations.

### Spatial and Temporal Variability

The microbial community composition of well 5 shows to be stable throughout the investigation period. A steady diversity index together with constant cell counts indicates little influence of surface water microorganisms. Contamination is likely to decrease species richness, thus high diversity parameters can indicate environmental quality (Covich et al., 2004). As this well is located further away from the alluvial surface water than others, it is most likely that increased distance prolongs filtration processes of surface water, leading to decreased influence of allochthonous organisms in well water (Hiscock and Grischek, 2002). These results are in accordance with the findings from Lee et al. (2018) who showed microbial community shifts in groundwater closer to the adjacent stream rather than in those parts with some distance from stream water. Furthermore, due to hydrological conditions, local groundwater recharge might contribute more strongly to the microbial and chemical composition of this well (Hoehn and Scholtis, 2011), thus minimizing inflow of surface water at this point. The latter is supported by an elevated electrical conductivity as compared to other wells, as in many cases groundwater is characterized by a higher mineral content than surface water (Sheets et al., 2002).

In contrast to that, well 4 shows a strong influence induced by temporal variation, leading to major shifts in diversity parameters from April 7 to May 3. Increased cell counts suggest that allochthonous microorganisms strongly affect microbial community compositions in abstraction wells due to reduced interstitial residence time of surface water, which is known to be a critical factor in terms of allochthonous microbial loads (Hiscock and Grischek, 2002).

### Estimated Absolute Abundance (EAA)

Absolute quantification of relative abundances gives a surplus value on the generated information on microbial dynamics such that conclusions can be drawn on actual shifts in microbial populations (Props et al., 2017). In general, mixing surface water with groundwater does increase cell counts due to cell introduction from surface water, the stimulation of heterotrophic respiration, as well as altered organic carbon compositions and is therefore used to monitor microbial dynamics (Stegen et al., 2016; Epting et al., 2018; van Driezum et al., 2018). The impression of suppressing microbial phyla due to the increase of others suggests that allochthonous microorganisms inhabit ecological niches instead of autochthonous ones (Props et al., 2017). Our results emphasize that with the infiltration of surface water rather new niches are created giving rise to changing microbial groups. This assumption is encouraged by the fact that microbial metabolic limitations are overcome by mixing of waters (Stegen et al., 2016) leading to elevated abundances of microbial taxa and that allochthonous contaminations occur temporarily and are unlikely to persist (Pronk et al., 2009). However, on family level diverse shifting patterns within phyla are observed: (i) consistent families and rise of new ones (Verrucomicrobia), (ii) increase of existing families (Bacteroidetes), (iii) suppression of families in favor of others (Proteobacteria), and (iv) new development with no or very little previous abundance of the overall phylum (Actinobacteria and Armatimonadetes). The differences in phylum and family shifting patterns indicate that phylogenetic similarity among bacterial groups can lead to replacement of organisms in certain niches, possibly due to functional similarities (Petchey and Gaston, 2006).

### Microbial Shifting Patterns

Our investigations suggest that impacts on microbial communities after a flood event or increased water abstraction in wells do not occur immediately, but after a certain period of time. The combination of increased surface water levels and decreased well water levels results in a higher probability of shifting microbial communities. Most likely, not so much the increase of water level differences, but rather the changes in differences due to abstraction regimes contribute to different water compositions. These findings are in accordance with results from Ascott et al. (2016), who observed rapid recovery of water quality with increased abstraction rates and slower recoveries due to temporary reductions in abstraction rates after extreme flooding. Nevertheless, the delay in microbial shifts may be a result of residence time of water in the subsurface area. Therefore, major shifts in microbial community compositions from April to May during this study may derive from diverse abstraction regimes together with previous rainfalls, but dynamic hydraulic conditions in the river bed make predictions on time shifts difficult (Hiscock and Grischek, 2002). The immediate investigation of a flood event in May did not show major shifts in community compositions, although well 6 shows slight changes toward community compositions of wells 1 – 4, giving rise to expect similar dynamics in this well as a consequence of proceeding rain events and hydraulic differences.

### Taxonomy

The overall findings of taxonomic groups are in accordance with other data: Graham et al. (2017) compared microbial communities of Inland, Nearshore and River water. All of the ten most abundant phyla of each sample group were also identified in our investigation, additionally we found the phyla Rokubacteria and Patescibacteria. The shifts from our results are roughly in accordance with their findings in River water as compared to Inland and Nearshore samples, respectively: Bacteroidetes, Actinobacteria and Verrucomicrobia are together with Proteobacteria among the most abundant phyla in River water samples, Armatimonadetes only occur in River samples and Cyanobacteria that are not abundant in Inland samples and little abundant in Nearshore samples show abundancies of 4.66% in River samples. Findings from Lee et al. (2018) show similar results. In our study, the four strongest increasing phyla, i.e., Verrucomicrobia, Actinobacteria, Cyanobacteria, and Bacteroidetes, are all considered typical freshwater bacteria (Newton et al., 2011) and have also been found previously in freshwater by Iliev et al. (2017) and Kim et al. (2018) and others. Patescibacteria were first reported in groundwater and sediments of anoxic environments (Elshahed et al., 2005; Youssef et al., 2011; Wrighton et al., 2012), but showed to be widespread in most semiaquatic habitats (Sánchez-Osuna et al., 2017). Rokubacteria, also found in groundwater samples, are most closely related to the phylum Nitrospira. They are characterized by a high level of genetic heterogeneity and show potential for a versatile, mixotrophic metabolism. Nevertheless, they are rather low in abundance at different sites, possibly explaining the lack of identification in other studies (Becraft et al., 2017).

Actinobacteria are abundant in rivers (Zwart et al., 2002), although the family Sporichthyaceae, which contributes most to the increase in Actinobacteria has been found in soils (Tamura, 2014), suggesting that entry occurs via the subsurface area rather than surface water itself. Typically, Actinobacteria are very small (<0.1 μm^3^), free living cells (Hahn et al., 2003). In contrast, Bacteroidetes are mainly particle associated and strongly dependent on the availability of organic matter (Newton et al., 2011). They are able to digest large organic polymers (Gómez-Pereira et al., 2010; McBride, 2014) and the most abundant family among freshwaters representing Flavobacteriaceae (Gómez-Pereira et al., 2010) was also found in our investigations. Cyanobacteria are associated with algal blooms in surface water (Berry et al., 2017) and may produce toxins that affect drinking water quality (Walter et al., 2018). Due to their dependency on a light source, increase happens due to intrusion rather than growth and persistence is very unlikely. The abundance of Verrucomicrobia has been underestimated in the past due to poor primer coverage (Bergmann et al., 2011). However, they are present in soil and freshwater habitats accounting for up to 42% in lake samples (Chiang et al., 2018). All Verrucomicrobia families found during this investigation were also abundant elsewhere in river water (Balmonte et al., 2016). They show diverse metabolic capabilities among clades (Chiang et al., 2018), suggesting a high versatility and capability of persisting different environmental conditions.

Heatmap grouping results underline the existence of well adapted autochthonous microbial populations and typical shifts from one composition to another as a consequence of allochthonous microbial influences, as it was previously suggested by Danielopol et al. (2003) and Griebler and Lueders (2009) and others. The similarity among samples at the beginning of the investigation and subsequent divergence of well microbial characteristics point to the cumulative outcome of diverse biotic and abiotic impacts leading to differences in well water characteristics.

### Practical Implications

The occurrence of surface-groundwater mixes is subject to temporally dynamic and spatially complex processes, making predictions among them difficult (Stegen et al., 2016). The concept of detecting mixtures of surface water and groundwater via microorganism shifts rather than studying distribution patterns of water types within the passage through the subsurface area can provide adequate statements about mixing patterns in the alluvial groundwater. Up to now, no consensus exists about typical pristine groundwater bacteria (Pernthaler, 2013) and legislations lack microbial parameters as indicators of groundwater quality (European Commission, 2000), thus no definition of “good” microbial groundwater quality exists. Our results give a comprehensive insight into microbial communities from a river bank filtrate used for drinking water and allow for conclusions on the spatial distribution of bank filtrated surface water among wells. Identified microbial dynamics in dependency of water levels resulting from both rain events as well as varying pumping rates can be indicative regarding the improvement of a constant overall microbial water composition in the subsequent drinking water distribution system. Additional microbial investigations of river water could determine the origin of allochthonous microorganisms as it was performed by Kim et al. (2018) and Lee et al. (2018). Furthermore, abiotic factors are required to better understand the mechanisms of changing water qualities. Adapting operational regimes based on the revealed information about microbial shifts together with abiotic parameters, such as electrical conductivity, oxygen content and temperature, shows potential to maintain a microbiologically stable raw water and thus minimizes the risks of unexpected technical as well as hygienic disturbances. It is known that allochthonous contaminations as a consequence of increased water levels and shortened residence time in the river bank are unlikely to persist in the groundwater (Pronk et al., 2009). Nevertheless, a large proportion of VBNC microorganisms give rise for monitoring microbial community compositions in abstraction wells (Ramamurthy et al., 2014). In order to determine how long communities will need to return to their initial conditions and what factors influence the recovery (Ascott et al., 2016), investigations implemented on a longer time-span are required. Furthermore, online monitoring of cell counts in wells can indicate adequate timing of sampling for sequencing analyses, as suggested by Besmer et al. (2016). Although the dynamics in microbial community compositions outline water mixture patterns, the consequences introduced microorganisms might have on water regarding downstream drinking water treatment and distribution are widely unknown and need to be further investigated.

## Data Availability Statement

The dataset analyzed for this study can be found in the European Nucleotide Archive (ENA) under the accession number PRJEB28172. The flow cytometry data can be found on the flowrepository database (https://flowrepository.org) under the ID number FR-FCM-ZYRK. The analysis pipeline is available on github under the repository link chrischoen/r.riverbankfiltrate.

## Author Contributions

CF carried out sample collecting, laboratory work, data analysis, and drafted the manuscript. CS, PP, and DK implemented data analyses and helped to draft the manuscript. EM, MZ-P, and RP conceived the study and revised the paper. KD provided facilities and helped implementing the laboratory work. All authors have approved the submission of the article.

## Conflict of Interest Statement

The authors declare that the research was conducted in the absence of any commercial or financial relationships that could be construed as a potential conflict of interest.

## Footnotes

1 https://qiime2.org [access 12/06/2018]
