# Supplementary material for: Assessment of Microbial Community Dynamics in River Bank Filtrate Using High-Throughput Sequencing and Flow Cytometry
Source: Front Microbiol. 2018 Nov 29;9:2887. doi: 10.3389/fmicb.2018.02887 (PMC6281747; doi:10.3389/fmicb.2018.02887)
Supplement: Supplementary file 1 [file Data_Sheet_1.docx]

Supplementary Material

Assessment of microbial community dynamics in river bank filtrate using high-throughput sequencing and flow cytometry

Christina J. Fiedler, Christoph Schönher, Philipp Proksch, David Johannes Kerschbaumer, Ernest Mayr, Marija Zunabovic-Pichler^*^, Konrad J. Domig, Reinhard Perfler

*** Correspondence:** Dipl.-Ing. Dr. Marija Zunabovic-Pichler: marija.zunabovic@boku.ac.at

**Captions of the Supplementary Figures (S1-S6)**

Supplementary Figure 1. Exemplary scatterplot of two fluorescence parameters (FL1-A and FL3-A) to visualize the gating strategy. Each point represents a detection. Cells are separated from (in)organic noise by means of a digital gate (red) uniformly applied to all datasets.

Supplementary Figure 2. Relative abundances of phyla at the respective dates (cut-off value 1%). Reads from triplicates were pooled to one sample each.

Supplementary Figure 3. Selected results of DAtest between wells 1 – 3 on April 7 and May 3, relative abundances.

Supplementary Figure 4. Selected results of DAtest between wells 1 – 3 on April 7 and May 3, absolute abundances.

Supplementary Figure 5. Temperature course of wells and surface water. W1 – 6, wells 1 – 6.

Supplementary Figure 6. Course of electrical conductivity within wells and surface water. W1 – 6, wells 1 – 6.

**Supplementary Figures 3 and 4 – additional information**

DAtest was used for the assessment of suitability of various differential abundance testing procedures (for a current list see https://github.com/Russel88/DAtest) for the dataset of interest (52 phyla, 6 samples, 2 groups comprising the same wells 1-3). Essentially, the following steps have been performed:

1. Taxonomic agglomeration of sequence data (combined as phyloseq object including sample data, a table of sequence variants and a taxonomy table) on phylum level using phyloseq’s tax_glom function
2. Transformation of raw counts to relative abundances and subsequent multiplication with flow cytometric total cell counts resulting in estimated absolute abundances (EAAs)
3. Removal of low abundance phyla with the function preDA of package DAtest (parameters: min.samples=0, min.reads=1, min.abundance=0.0001). This resulted in a reduction of the number of phyla from 52 to 36.
4. Application of the function testDA of package DAtest for a combination of different input parameters, namely:
   1. spiking patterns (k) as the number of spiked features in each abundance tertile (lower, mid, upper): (1,1,1), (2,2,2), (3,3,3), (4,4,4), (5,5,5) and (6,6,6)
   2. effect sizes (effectSize): 2, 4, 8, 16, 32, 64, 128

The number of times to run the test was set to 100. By setting the parameter *relative* to true or false the calculations were performed with or without relative abundances. Setting *relative* to false (for absolute abundances) results in the in the deactivation of internal normalizations and methods that rely on such normalizations. As predictor the date of sampling was chosen. The parameter *paired* was defined as the name of the well. Not all of the methods provide the possibility of performing a paired analysis, thus only a subset of all available methods had been used.

For the assessment of the results plots of effect size versus DAtest’s score combined with different spiking patterns were produced with ggplot2 (Wickham, 2016) and are shown for a selection of methods in Figure S1 and Figure S2. The score is calculated as the (AUC-0.5) * Spike.detect.rate – FDR, with AUC being the area under the ROC curve and FDR being the false discovery rate (maximum score is 0.5).
