# Supplementary figures and images for: Assessment of Microbial Community Dynamics in River Bank Filtrate Using High-Throughput Sequencing and Flow Cytometry
Source: Front Microbiol. 2018 Nov 29;9:2887. doi: 10.3389/fmicb.2018.02887 (PMC6281747; doi:10.3389/fmicb.2018.02887)

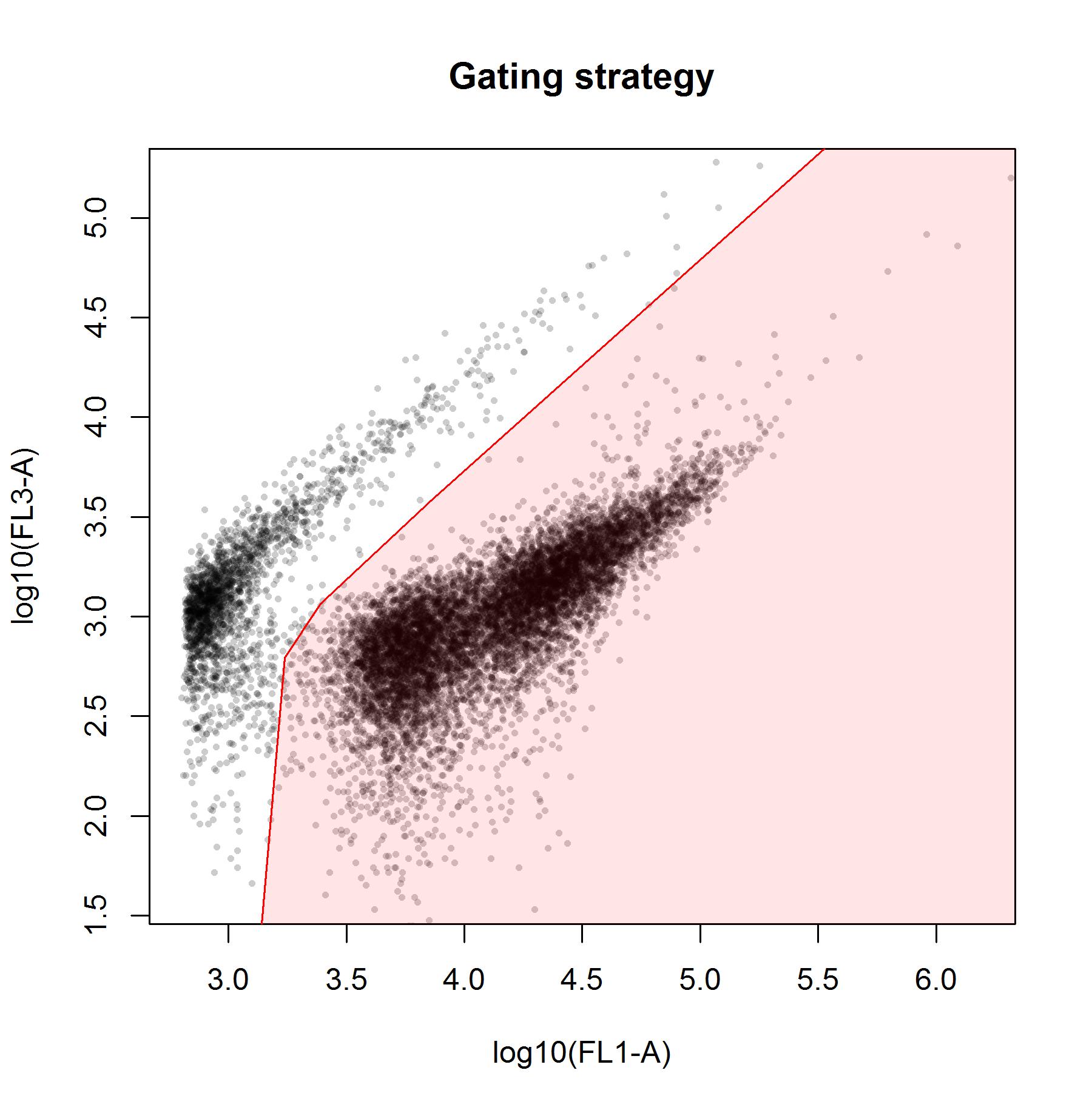

Supplement: Supplementary file 2 [file Image_1.jpeg]

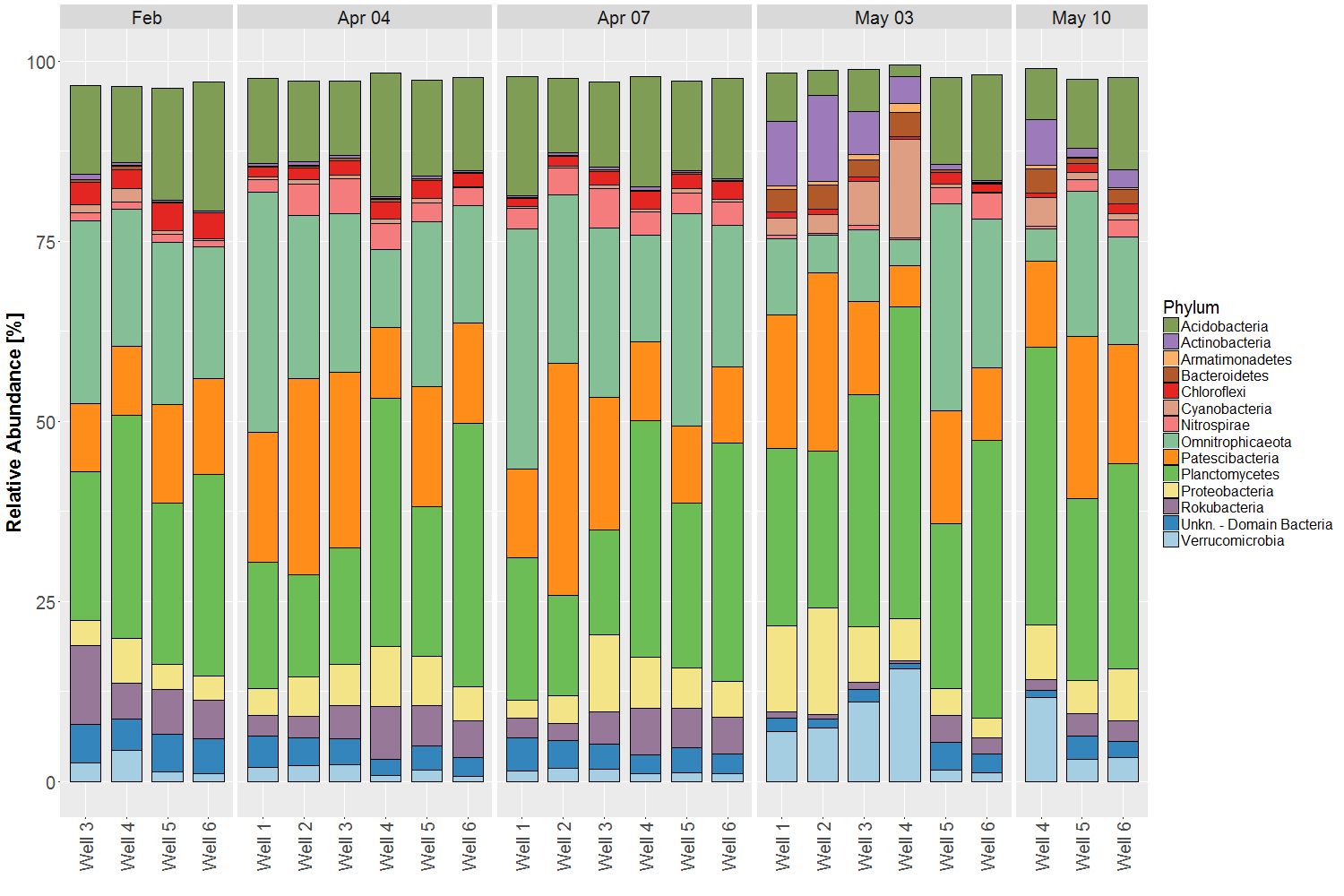

Supplement: Supplementary file 3 [file Image_2.jpeg]

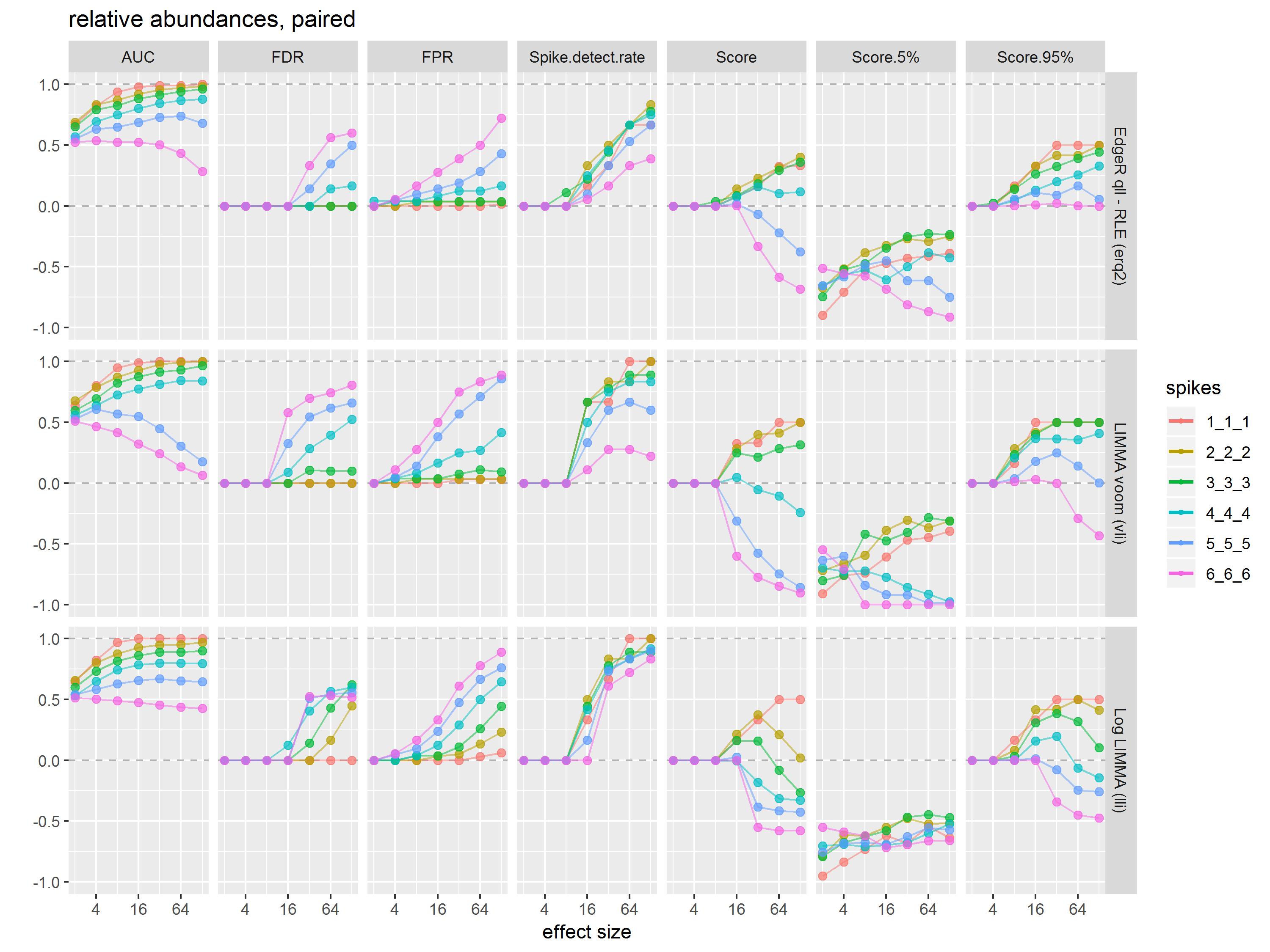

Supplement: Supplementary file 4 [file Image_3.jpeg]

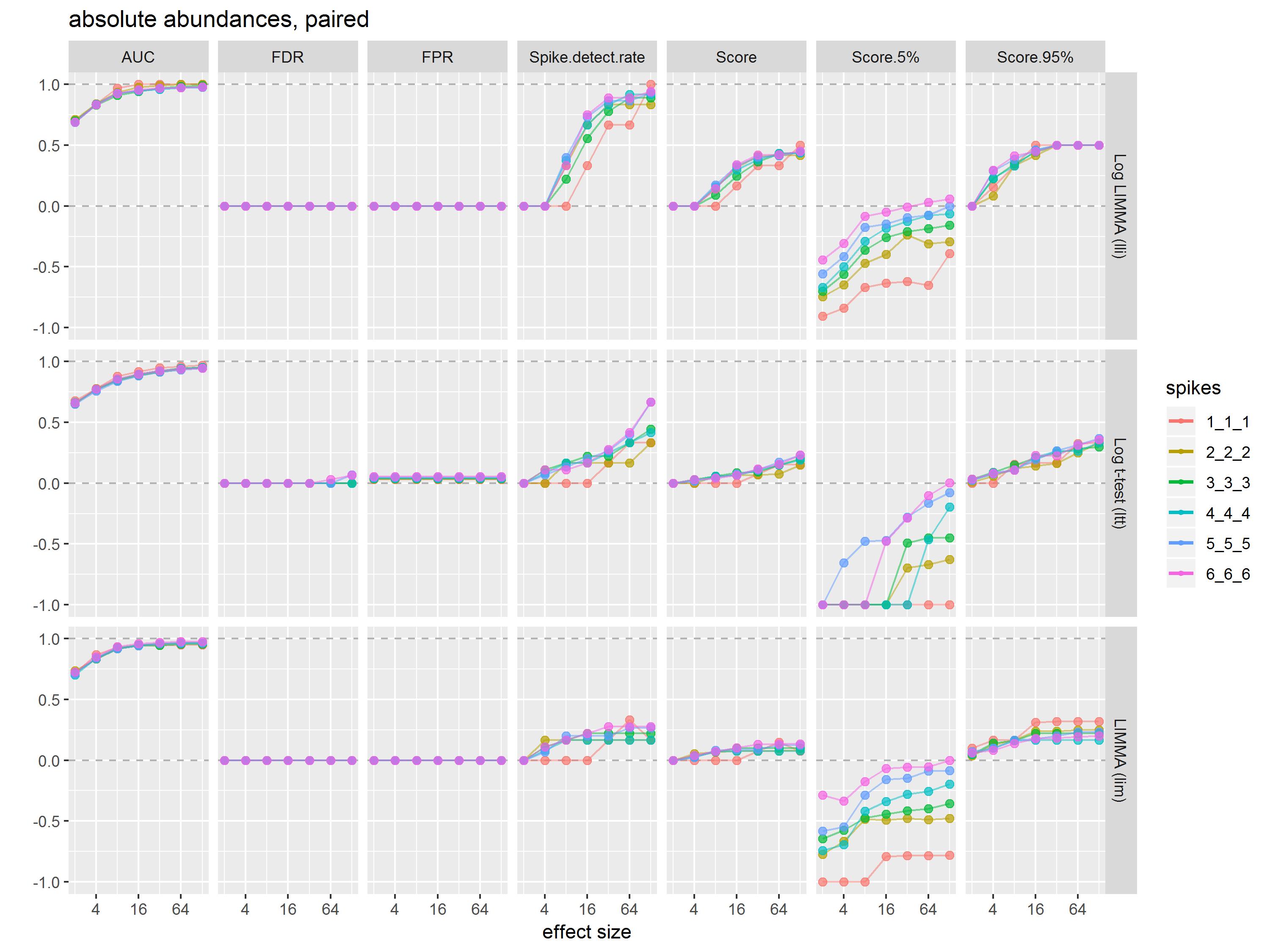

Supplement: Supplementary file 5 [file Image_4.jpeg]

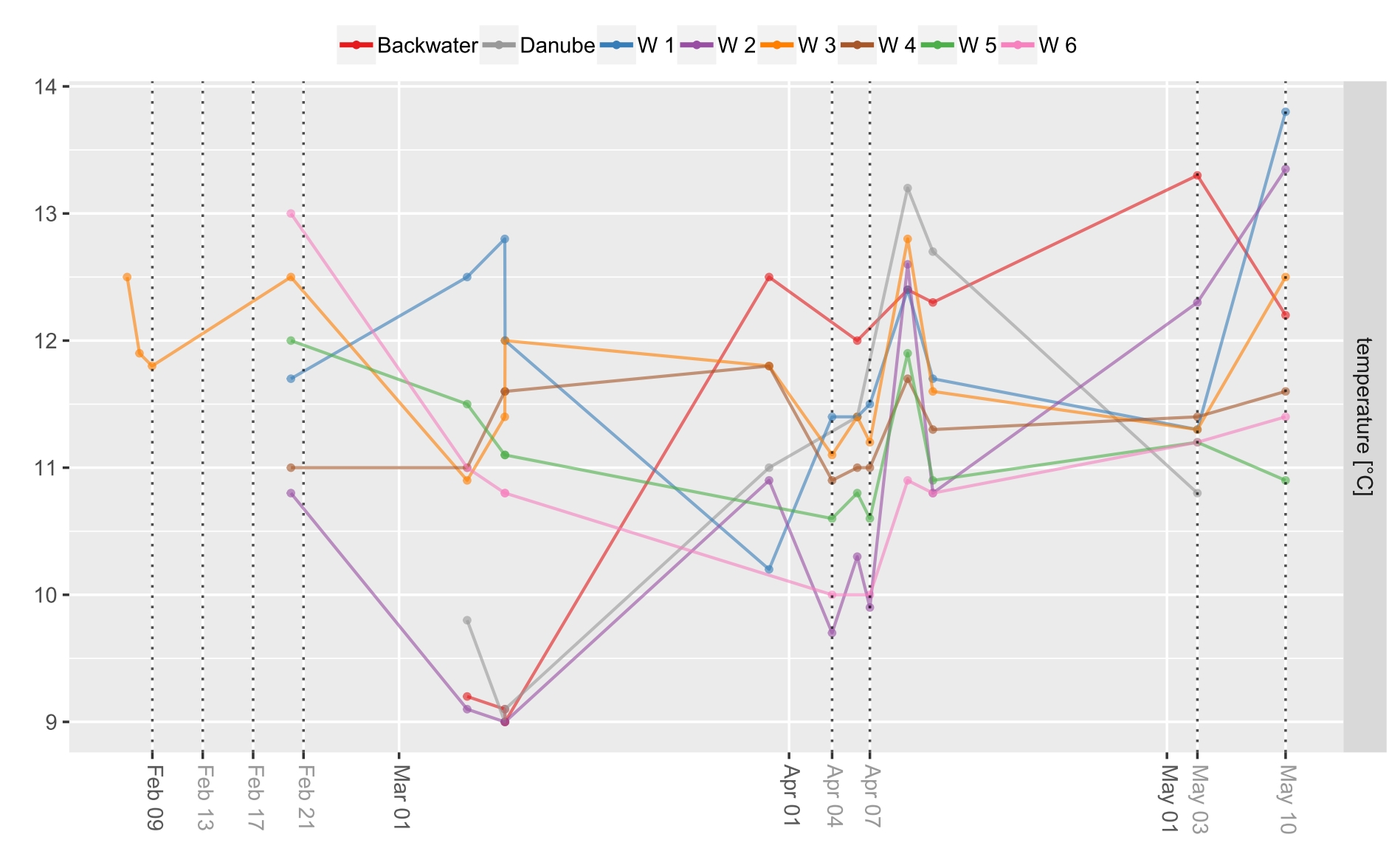

Supplement: Supplementary file 6 [file Image_5.jpeg]

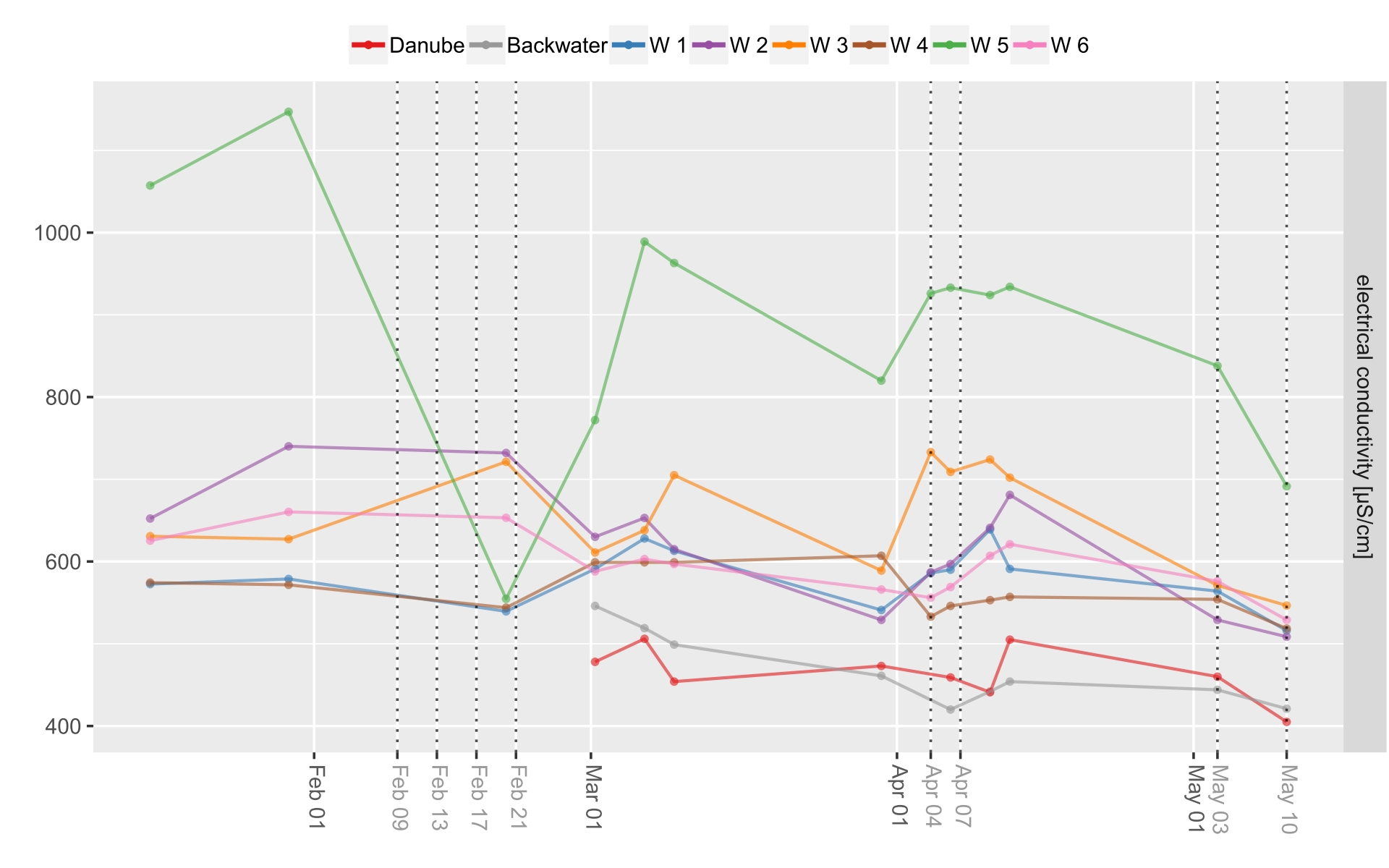

Supplement: Supplementary file 7 [file Image_6.jpeg]
